# The Effect of Intraoperative Hypothermia on Anastomotic Leakage After Esophagectomy

**DOI:** 10.3390/cancers17071166

**Published:** 2025-03-30

**Authors:** Lorenzo Cinelli, Stefano Turi, Francesco Puccetti, Yong-Ha Lee, Riccardo Rosati, Ugo Elmore, on behalf of the OSR CCeR Collaborative Group

**Affiliations:** 1 Department of Gastrointestinal Surgery, IRCCS San Raffaele Scientific Institute, 20132 Milan, Italy; cinelli.lorenzo@hsr.it (L.C.); yongha92@gmail.com (Y.-H.L.); rosati.riccardo@hsr.it (R.R.); elmore.ugo@hsr.it (U.E.); 2 Department of Anesthesia and Intensive Care, IRCCS San Raffaele Scientific Institute, 20132 Milan, Italy; turi.stefano@hsr.it; 3 School of Medicine, Vita-Salute San Raffaele University, 20132 Milan, Italy

**Keywords:** esophagectomy, anastomotic leakage, hypothermia, core temperature, Ivor Lewis

## Abstract

Background/Objectives: Inadvertent intraoperative hypothermia is a common occurrence during major surgery, and some preclinical studies have reported its association with postoperative anastomotic leakage (AL). In the present study, we demonstrated the correlation between intraoperative hypothermia and postoperative outcomes after Ivor Lewis (IL) esophagectomy for cancer. Methods: Retrospectively, patients were divided into three groups on the basis of their time-weighted average temperatures (TWA): normothermia (N-TWA 36–37.5 °C), mild hypothermia (M-TWA 35–36 °C), and severe hypothermia (S-TWA < 35 °C). Results: Starting from 254 consecutive patients, 95 were included in the final analysis, classified according to intraoperative temperature: S-TWA = 19 (20%), M-TWA = 62 (65.3%), and N-TWA = 14 (14.7%). S-TWA was related to lower BMI (*p* = 0.001), diagnosis of squamous cell carcinoma (*p* = 0.029), and shorter operation times (*p* = 0.006). In the same way, AL was more related to S-TWA when compared with M-TWA and N-TWA (31.6% vs. 6.5% vs. 14.3%, *p* = 0.015). After multivariate analysis, S-TWA remained as the only predictive factor of AL (OR 5.385, 95%CI 1.502; 19.310; *p* = 0.010). Instead, higher BMI was found to be a protective factor for S-TWA (OR 0.818, 95%CI 0.723; 0.926: *p* = 0.001). Conclusions: S-TWA seems to be a major independent risk factor for AL after IL esophagectomy. Implementation of perioperative measures, aimed to prevent severe intraoperative hypothermia, could potentially be crucial to improve surgical outcomes.

## Simple Summary

Intraoperative hypothermia is a common issue during major surgery, and preclinical studies have demonstrated how it impairs tissue oxygenation, wound healing, and immune response. However, the detrimental effects of low core body temperature have never been demonstrated in esophageal cancer surgery. This is the first study exploring the correlation between intraoperative hypothermia and postoperative anastomotic leakage after esophagectomy. The reported findings suggest the relevance of maintaining normal body temperature in improving postoperative outcomes following esophageal resections.

## 1. Introduction

Esophageal cancer is the eighth most common tumor and the sixth leading cause of cancer-related deaths [1]. Despite advances in multimodal therapy, surgery remains the curative treatment for resectable esophageal cancer, combined with chemo- or chemoradiotherapy according to the clinical and pathological stage [2]. The terms of esophageal resections, both the surgical approach and technique, are based on tumor and individual parameters, and Ivor Lewis (IL) esophagectomy represents the most prevalent procedure in Western countries, being routinely indicated for tumors of the lower and junctional esophagus [3].

Postoperative recovery after esophagectomy is burdened by the potential development of procedure-related complications, with 90-day mortality rates of 2.5–7% and high morbidity rates [4,5]. Anastomotic leakage (AL) is one of the most life-threatening complications after IL esophagectomy, negatively impacting survival, length of hospital stay, resumption of oral feeding, readmission rate, long-term quality of life, healthcare costs, and resource utilization. In 2022, the International Esodata Study Group (IESG) reported the clinical benchmarks of postoperative morbidity after esophagectomy, including AL rates of 13.1%, with need for reintervention in 3.0% [6]. Despite the growing literature on the burden of AL, its development remains unpredictable and poorly understood. Several factors have been described to explain the multifactorial origin of AL, including local (i.e., perfusion of the gastric conduit), technical (i.e., type and location of anastomosis), general (i.e., demographics and medical conditions), tumoral (i.e., stage and neoadjuvant treatment), and hemodynamic (i.e., hypotension, blood loss, venous congestion, respiratory dysfunction) predictors [7]. Comprehensive knowledge of specific and general predictive factors is crucial to anticipate the risk of AL, leading to optimized perioperative procedures and tailored postoperative surveillance.

Inadvertent intraoperative hypothermia is a common occurrence during major surgery as a consequence of general anesthesia and the prolonged length of operations. It has been recognized that perioperative hypothermia causes delayed post-anesthetic recovery and increases surgical complications, such as wound healing and surgical site infections [8]. In particular, hypothermia triggers vasoconstriction and reduces tissue oxygenation and tissue healing [9].

Some preclinical studies have reported its effect on the suppression of inflammatory response, fibrotic reaction, and collagen deposition, and on the increased production of reactive oxygen species (ROS), describing a detrimental effect on the healing of intestinal anastomoses [10,11]. However, its direct impact on AL after esophagectomy has not been clinically described yet.

The present study aimed to assess the correlation between intraoperative hypothermia and short-term postoperative outcomes after IL esophagectomy for cancer, with a special focus on AL.

## 2. Materials and Methods

The present study was conducted in accordance with the STrengthening the Reporting of OBservational studies in Epidemiology statement (STROBE) guidelines (Table S1) [12].

### 2.1. Study Design

All the patients who consecutively underwent IL esophagectomy between January 2020 and February 2022 at San Raffaele Hospital (Milan, Italy) were retrospectively analyzed from an IRB-approved prospectively maintained institutional database, according to the following inclusion criteria.

- Histologically confirmed diagnosis of adenocarcinoma or squamous cell carcinoma (SCC) of the medium and distal third of the esophagus, with clinical eligibility for surgery (any T, any N, M0); IL esophagectomy regardless of the surgical approach (i.e., either laparoscopic, open or hybrid);
- Aged ≥ 18 years;
- Any multimodal strategy (i.e., either neoadjuvant therapy or upfront surgery);
- Standardized management, including perioperative multidisciplinary assessments and the ERAS-based clinical protocol [13];
- Full availability of electronic records regarding intraoperative temperature.

The exclusion criteria were any histological subtypes other than adenocarcinoma or SCC, any resection types other than IL esophagectomy, and multivisceral resections.

Demographic variables (age, gender, body mass index (BMI), age-adjusted Charlson comorbidity index (aa-CCI) [14], American Society of Anesthesiologists (ASA) score [15]) and oncological data (details of neoadjuvant and surgical treatments and diagnosis) were also collected.

Total minimally invasive IL esophagectomy (laparoscopic and thoracoscopic) represented the surgical approach of choice unless preoperative findings of technical limitations (i.e., suspicion of extra-esophageal infiltration, lymph node spread to distant sites, or bulky dimensions of the primary tumor) or patient-related impediments for the minimally invasive technique were encountered. Patients who required conversion from laparoscopy or thoracoscopy to the open approach were included in the minimally invasive group according to the intention-to-treat principle. Hybrid IL esophagectomy (laparoscopy and thoracotomy) was included among the open procedures due to the significant impact of the thoracic stage on body temperature, given the length and exposure of the lungs, heart, and greater vessels. Conversely, other hybrid combinations (laparotomy and thoracoscopy) did not appear in the present study series.

### 2.2. Surgical Procedure

The first phase of the operation consisted of the abdominal stage. After performing the Kocher maneuver, a pyloromyotomy with pyloroplasty was realized to prevent postoperative delayed gastric emptying. The stomach was prepared by preserving the right gastroepiploic and pyloric vessels for its vascular supply, and a modified D2 lymphadenectomy was completed. At this time, adequate perfusion of the gastric conduit was assessed through a near infrared camera after an intravenous bolus of 0.3 mg/kg of indocyanine green (ICG) (Verdye, Diagnostic Green GmbH, AschheimDornach, Germany). Thereafter, the gastric conduit was fashioned. During the thoracic stage, after completion of esophagectomy and mediastinal lymphadenectomy, the gastric conduit was pulled up via the posterior mediastinal route. Another perfusion evaluation via ICG angiography using the same protocol as in the abdominal stage was performed in the thorax, soon before fashioning the anastomosis. A purse-string stapled circular end-to-side esophagogastric anastomosis was the routine technique. A reinforcement of the pleura was applied, if feasible. The fully detailed surgical procedure was reported in previously published articles by our group [16,17].

### 2.3. Perioperative Standardized Protocol

A standardized enhanced recovery perioperative pathway [18] was institutionally applied to the whole series, embedding all types of surgical approach (Figure S1). General anesthesia induction was performed by administering fentanyl (1 mcg/kg), propofol (2 mg/kg), and rocuronium (0.6 mg/kg). A double-lumen endotracheal tube was positioned to achieve one-lung ventilation (OLV), and a fiberoptic bronchoscope was used to confirm correct tube placement. The left radial artery and the central venous catheter were routinely positioned. Intraoperative fluid administration was managed using a goal-directed therapy strategy, through a mini-invasive Flo-trac system (Edwards Lifesciences, Irvine, CA, USA), with a target MAP of 70 mmHg and continuous measurement of stroke volume (SV) and stroke volume variations (SVV) [19,20]. According to a lung-protective ventilatory management, a tidal volume of 6–8 mL/kg was chosen during the abdominal phase and then reduced to 4–6 mL/kg during OLV, associated with a PEEP of 5–7 cmH2O. A paravertebral catheter for postoperative pain control was positioned at the end of the thoracic phase under direct vision by the surgeons, as previously described by Yamauchi et al. [21]. Multimodal analgesic therapy with acetaminophen at fixed intervals and non-steroidal anti-inflammatory drugs (NSAIDs) at the patient’s request were also prescribed. After adequate monitoring in a dedicated recovery room, all patients were transferred to the intensive care unit or general ward on the basis of clinical judgment. Postoperatively, patients were managed using a standardized therapeutic protocol for postoperative hypotension with a target MAP value of 70 mmHg, as suggested by Klevebro et al. [22]. Pain intensity and possible side effects related to analgesic techniques were monitored by a dedicated acute pain service [23].

In our clinical practice, the patients’ core temperature was continuously monitored using a temperature-sensing urinary catheter during surgery and was automatically recorded at 5 min intervals. A forced-air warmer (Bair Hugger model 505, Augustine Medical, Cape Town, South Africa) was used to maintain normothermia during surgery in all patients. Prewarmed fluids or intravenous (IV) fluid warming systems (Smiths Medical Hotline HL-90 Blood and Fluid warmer, Icumedical, San Clemente, CA, USA) such as a low-flow anesthesia system (Flow-e Anesthesia Machine, Getinge, Rastatt, Germany) were also used. Preoperative warming was not performed. The operation room’s temperature was maintained constant (22 °C).

### 2.4. Definition of Outcomes

Short-term surgical and anesthesiologic outcomes (intra- and postoperative) were analyzed. The series was classified into the following three groups on the basis of the patients’ intraoperative time-weighted average (TWA) core temperature: normothermia (36.0 to 37.5 °C), mild hypothermia (35.0 to <36.0 °C), and severe hypothermia (<35 °C). Starting from the thermal curve of each patient, the TWA temperature was calculated as the area under the curve divided by the duration of monitoring [24]. Intraoperative blood loss was defined as the amount of blood suctioned during the operation.

The type and severity of postoperative complications and AL were assessed according to the Clavien–Dindo and the Esophagectomy Complications Consensus Group (ECCG) classifications [25,26]. The clinical suspicion of AL was confirmed by a CT scan of the chest and abdomen with oral contrast swallow, endoscopy, or surgery. Surgical site infections (SSI) included infection of surgical wounds, intra-abdominal fluid collection, and sepsis. Delayed gastric conduit emptying (DGCE) was determined after radiographic, symptomatic, or pharmacologic evidence of a delay [27,28,29]. Overall complications also included either in-hospital or 90-day mortality, while readmission was recorded within 30 days of surgery.

### 2.5. Statistical Analysis

Categorical data were expressed as the number of patients and percentages. The normality of the distribution of continuous variables was tested by visual inspection of the histograms and one-sample Kolmogorov–Smirnov tests. Continuous variables with a normal distribution were presented as the mean ± standard deviation (SD). For continuous non-normal variables, the median and interquartile range (IQR) were reported. Comparisons between groups were performed using the Chi-square test or Fisher’s exact test, as appropriate, for categorical data. Continuous variables were compared using Student’s *t* test or the Mann–Whitney U test, as appropriate. Bonferroni correction was applied to account for multiple testing when necessary. Univariate and multivariable logistic regression analyses were performed in order to identify predictors of anastomotic leakage. The final multivariable models were obtained via backward variable selection. Variables that were statistically significant in the univariate analysis were included in the final multivariable models, which were also adjusted for several baseline confounders (gender, age, and neoadjuvant treatment). The final multivariable model assessing the determinants of severe hypothermia was also corrected for intraoperative blood loss, total intraoperative fluids, and operation time, as associations between these variables and body temperature have been previously reported in the literature [30,31].

Two-tailed *p*-values were considered significant when less than 0.05. Statistical analyses were performed by using IBM SPSS Statistics v27.0 (IBM Corp. Armonk, NY, USA).

## 3. Results

### 3.1. Study Population

During the study period, 254 patients underwent IL esophagectomy for cancer. After excluding incomplete electronic records regarding intraoperative temperature (*n* = 159), a total of 95 patients were considered for the present study. On the basis of the intraoperative TWA core temperature, 19 (20%) and 62 (65.3%) patients were classified as having severe and mild hypothermia, respectively. Intraoperative normothermia was reported in only 14 (14.7%) cases. The severe hypothermia group presented with a lower BMI (22.3 ± 3.7 vs. 24.6 ± 4 vs. 28.2 ± 5.4; *p* = 0.001) and shorter operative times (258 ± 47 vs. 280 ± 53 vs. 306 ± 45; *p* = 0.006) when compared with mild hypothermia and normothermia.

Moreover, patients with a diagnosis of SCC presented higher rates of severe hypothermia than those with adenocarcinoma (43.8% vs. 15.2%; *p* = 0.029). Regarding postoperative complications, AL was significantly higher in the severe hypothermia group as compared with the other groups (31.6% vs. 6.5% vs. 14.3%, *p* = 0.015). Comparisons among groups according to intraoperative core TWA temperature are reported in Table 1.

### 3.2. Characteristics of Groups According to Postoperative AL

BMI was significantly lower in the AL group as compared with patients without anastomotic complications (22 ± 2.3 vs. 25 ± 4.6; *p* = 0.012), while a diagnosis of SCC presented higher rate of AL when compared with adenocarcinoma (31.2% vs. 8.9%; *p* = 0.028). Moreover, AL was more related to severe hypothermia than intraoperative TWA temperature > 35 °C (31.6% vs. 7.9%; *p* = 0.013) (Table 2).

### 3.3. Analysis of BMI and Operative Times According to Pathological Features

Adenocarcinoma presented a longer duration of surgery (285 ± 53 vs. 255 ± 41; *p* = 0.016) and this difference remained statistically significant in the abdominal stage when compared with SCC (139 ± 50 vs. 114 ± 33; *p* = 0.046). However, no differences regarding the duration of the thoracic stage, the extension of lymphadenectomy, and BMI were found among groups (Table 3).

### 3.4. Predictors of AL and Intraoperative Severe Hypothermia

The final multiple regression analysis showed severe intraoperative hypothermia as the only independent predictive factor of AL [odds ratio (OR) 5.385, 95% confidence interval (CI) 1.502; 19.310; *p* = 0.010] (Table 4).

Instead, higher BMI was found to be a protective factor able to reduce intraoperative risk of severe hypothermia [OR 0.818, 95% CI 0.723; 0.926: *p* = 0.001] (Table 5).

## 4. Discussion

This study investigated the association between intraoperative hypothermia and AL in patients who underwent IL esophagectomy for cancer. Patients who experienced severe intraoperative hypothermia were demonstrated to be associated with a higher occurrence of AL. Recently, similar evidence has been previously reported by Ju et al., showing a relationship between intraoperative management of body temperature and the postoperative onset of pancreatic fistula following pancreaticoduodenectomy [30]. As far as we know, this study is the first to demonstrate the significant association of intraoperative hypothermia with AL in esophageal cancer surgery.

Several mechanisms may explain the detrimental effect of intraoperative hypothermia on the consolidation of esophagogastric anastomosis. The fashioning of the gastric conduit induces significant changes in the microcirculation of the stomach, promoting an inflammatory reaction up to the anastomotic region of the gastric fundus [32]. Extending to the perioperative period, hypothermia can be also considered responsible for thermoregulatory vasoconstriction, reducing tissue oxygen tension over time. Subsequently, this leads to the generation of ROS, which are negatively involved in the mechanism of tissue regeneration and impair the perioperative immune response required for proper intestinal anastomotic healing [33,34]. Given that intraoperative hypothermia is relatively common in major abdominal surgery and is potentially preventable, these findings hold significant clinical implications for preventing AL and improving the outcomes of patients after IL esophagectomy.

However, our findings demonstrated a significant failure rate of the existing arrangements for the prevention of intraoperative fluctuations in body temperature, drawing increasing attention to individual and preoperative features. Consistent with the current literature, our results indicated low BMI as a risk factor for perioperative hypothermia, highlighting the significant role of adipose tissue in reduced heat loss [35,36]. It is well established that low BMI is a prominent risk factor for SCC of the esophagus [37,38], whereas a high BMI is directly correlated with esophageal adenocarcinoma [39,40]. Nonetheless, the present series did not reveal any statistically significant differences between histological type and BMI (Table 3).

Interestingly, intraoperative hypothermia occurred more frequently during shorter operations and, although prolonged surgery has generally been associated with hypothermia, the present analysis suggested that normothermia requires time and precision to be achieved and maintained throughout esophagectomy [31,41].

A review by Sessler [9] suggests that temperature primarily decreases at the onset and during the first hour of general anesthesia due to anesthetic-induced vasodilation and the subsequent redistribution of body heat from the core to peripheral tissue. Subsequently, temperature gradually increases during surgery in actively warmed patients. Therefore, it may be argued that a shorter operative time does not allow enough time to increase body temperature in non-prewarmed patients, which is consistent with our findings. It is widely acknowledged that forced-air warming is more effective in maintaining normothermia during surgery compared with thermo-lite insulation [42,43,44]. The effectiveness of forced-air warming depends on the extent of the body surface area covered and effectively prevents perioperative hypothermia when the patient is in a supine position [45,46], while a smaller surface area is available in the lateral position typically adopted during thoracoscopy [47,48]. As a result, procedures with prolonged abdominal phase durations experience more sustained active warming, leading to more effective recovery of normothermia. This theory is further supported by our findings, wherein a diagnosis of SCC is more frequently associated with severe hypothermia.

In fact, within the context of standardized lymphadenectomy, abdominal lymph nodal pathological involvement is higher in cases of locally advanced adenocarcinoma of the esophagogastric junction compared with SCC of the distal esophagus. A recent multicenter study by Kurokawa et al. confirmed the higher rate of lymph node metastases in abdominal stations due to adenocarcinoma when compared with esophageal SCC [49]. This leads to prolonged abdominal stages during esophagectomy, allowing for more effective active warming.

An extensive part of the literature [7] has previously speculated on a large variety of perioperative factors that could be potentially associated with an increased risk of AL. Despite the multifactorial array of clinical determinants underlying the development of AL (i.e., visceral perfusion, level of esophagogastric anastomosis, anastomotic technique, age and preoperative comorbidities, cancer stage and neoadjuvant treatment, and institutional volume), the present analysis considered a homogenous series of esophageal cancer patients undergoing standardized perioperative care and surgical technique. Although stratification analyses were not performed, the lack of significantly different characteristics in the consecutively enrolled patients allowed a minimization of heterogeneity and possible effects of confounding factors. Many prior clinical studies have focused on preoperative or surgical factors in determining patients’ outcomes, while nonsurgical intraoperative factors have received limited attention [49,50]. However, significant physiological changes during general anesthesia could play a crucial role in gastrointestinal anastomotic healing, and improvements in anesthetic management may contribute to reducing the risk of significant surgical complications.

In our series, the surgical access did not influence the incidence of severe hypothermia, confirming scientific evidence indicating that laparoscopic techniques do not offer an advantage in maintaining body temperature compared with open surgery [51].

Furthermore, our findings, demonstrating a strong correlation between severe hypothermia and postoperative complications, are consistent with the results of the PROTECT trial [52]. In this international RCT, 5056 patients were randomized to receive aggressive intraoperative temperature management (target core temperature 37 °C) versus routine thermal management (target 35.5 °C). The incidence of a composite outcome, including myocardial infarction, surgical site infection, and the need for transfusion, was not different between groups. The PROTECT trial supported the hypothesis that maintaining a core temperature above 35 °C is sufficient to prevent major temperature-related complications.

This study has several limitations. First, the retrospective design may mislead some aspects of the present analysis and data interpretation. The study population belongs to a single high-volume center, and it can be questioned whether the results obtained in these patients can also be generalized to other institutions. Second, the patients presented different cancer subtypes (adenocarcinoma and SCC), undergoing either upfront surgery or neoadjuvant treatment. Also, preoperative chemotherapy or chemoradiotherapy were not equally administered, but rather depended on the disease and interpersonal characteristics of the study population. Third, evaluation of the core temperature was restricted to the intraoperative setting, preventing us from analyzing how further episodes of hypothermia could potentially affect mid-term outcomes. Therefore, extending monitoring to the first postoperative hours could shed light on possible management flaws or more effective interventions. Fourth, the limited sample size does not allow subgroup stratification or inferential analysis to strengthen the achieved results of the present study. Nonetheless, positives of the study lie in the consistency of management fundamentals, such as operative technique and the surgical team’s composition; standardized intraoperative assessment and perioperative care; and the considerable resources of a national referral center.

Fully aware of the complex and multifactorial development of AL after esophagectomy, our study offers a framework to investigate the potential roles of homeostasis-related intraoperative variables, significantly increasing the chances of enhanced postoperative outcomes and the definition of aggressive perioperative warming strategies in high-risk patients.

## 5. Conclusions

Transient nonsurgical intraoperative factors influence postoperative surgical outcomes. Severe intraoperative hypothermia was demonstrated to be an independent risk factor for AL after esophagectomy, which is potentially preventable through implementation of perioperative anesthetic management. Further studies involving multiple centers and randomized controlled trials with large sample sizes are required to confirm our results.

## Figures and Tables

**Table 1:** Differences between demographics and perioperative variables in patients from different intraoperative hypothermia groups.

	Severe Hypothermia	Mild Hypothermia	Normothermia	*p Value*
Patients	19 (20%)	62 (65.3%)	14 (14.7%)	
Intraoperative average temperature, °C *	34.6 ± 0.5	35.4 ± 0.3	36.2 ± 0.2	
Intraoperative TWA temperature, °C *	34.2 ± 2.2	35.4 ± 0.5	36.3 ± 0.3	
Preoperative				
Gender (male/female)	15/4	50/12	13/1	0.539
Age, years **	63 (56–71)	66 (60–73)	63 (51–72)	0.703
Body mass index, kg/m^2^ *	22.3 ± 3.7	24.6 ± 4	28.2 ± 5.4	0.001
ASA physical status III	10 (52.6%)	32 (51.6%)	8 (57.1%)	0.954
aa-CCI > 4	1 (5.3%)	12 (19.4%)	4 (28.6%)	0.215
Diagnosis				
Adenocarcinoma	12/79 (15.2%)	54/79 (68.4%)	13/79 (16.4%)	0.029
Squamous cell carcinoma	7/16 (43.8%)	8/16 (50%)	1/16 (6.2%)	
Neoadjuvant chemotherapy	17 (89.5%)	51 (82.3%)	10 (71.4%)	0.385
Neoadjuvant radiation therapy	8 (42.1%)	20 (32.3%)	2 (14.3%)	0.244
Intraoperative				
Minimally invasive/open approach	16/3	47/15	10/4	0.655
Operation time (min) *	258 ± 47	280 ± 53	306 ± 45	0.006
Intraoperative complications	4 (21%)	13 (21%)	2 (14%)	0.867
Blood loss, mL *	118 ± 38	147 ± 59	189 ± 97	0.567
Blood transfusions	0 (0%)	2 (3.2%)	1 (7.7%)	0.581
Infusions, mL *	2253 ± 954	2160 ± 1116	2650 ± 964	0.178
Intraoperative lactates (average) *	3.15 ± 1.85	3.21 ± 1.51	3.44 ± 1.58	0.723
Intraoperative lactates (highest value) *	4.42 ± 2.7	3.83 ± 1.78	4.5 ±2.1	0.549
Lymph node retrieval, n **	70 (61–85)	70 (61–81)	68 (62–90)	0.281
Postoperative				
Overall postoperative complications	15 (78.9%)	40 (64.5%)	10 (71.4%)	0.758
Severe complications (Clavien–Dindo ≥ 3a)	10 (52.6%)	27 (67.5%)	6 (42.8%)	0.649
Anastomotic leakage †	6 (31.6%)	4 (6.5%)	2 (14.3%)	0.015
Type I	1	0	1	
Type II	4	3	1	
Type III	1	1	0	
Delayed gastric conduit emptying	0 (0%)	2 (3.2%)	0 (0%)	1.000
Overall respiratory complications	7 (36.8%)	30 (48.4%)	5 (35.7%)	0.564
Pneumonia	2 (10.5%)	6 (9.7%)	1 (7.1%)	1.000
Cardiovascular complications	4 (21%)	10 (16.1%)	1 (7.1%)	0.546
Surgical site infections	4 (21%)	16 (26.1%)	3 (21.4%)	0.940
ICU stay	3 (15.8%)	8 (12.9%)	3 (21.4%)	0.831
Postoperative WBC, ×10^9^/L *	7.2 ± 1.8	6.6 ± 2.3	8.1 ± 3	0.263
Postoperative hemoglobin, g/dL *	12.4 ± 1.2	12.4 ± 1.7	12.6 ± 1.5	0.908
Postoperative lactates, mmol/L *	2.07 ± 1.40	2.15 ± 0.94	2.52 ± 2.22	0.382
In-hospital readmission	4 (21%)	19 (30.6%)	3 (21.4%)	0.663

* Average ± standard deviation; ** median (interquartile range); † According to the ECCG (Esophagectomy Complications Consensus Group). TWA, time-weighted average; aa-CCI, adjusted age-adjusted Charlson Comorbidity Index; ASA, American Society of Anesthesiologists; ICU, intensive care unit; WBC, white blood cells.

**Table 2:** Differences in demographic and perioperative variables between patients when considering anastomotic leakage.

	Anastomotic Leakage		
	Yes	No	*p Value*
Patients	12/95 (12.6%)	83/95 (87.4%)	
Preoperative			
Gender (male/female)	8/4	68/15	0.634
Body mass index, kg/m^2^ *	22 (±2.3)	25 (±4.6)	0.012
Age, years **	69 (55–80)	65 (59–75)	0.295
ASA physical status III	7 (58.3%)	43 (51.8%)	0.763
aa-CCI > 4	4 (33.3%)	13 (15.7%)	0.218
Adenocarcinoma	7/79 (8.9%)	72/79 (91.1%)	0.028
Squamous cell carcinoma	5/16 (31.2%)	11/16 (68.8%)	
Neoadjuvant chemotherapy	9/12 (75%)	3/12 (25%)	0.687
Neoadjuvant radiation therapy	3 (25%)	27 (32.5%)	0.747
Intraoperative			
Minimally invasive/open approach	10/12 (83.3%)	2/12 (16.7%)	0.726
Operation time (min) *	269 ± 51	281 ± 53	0.433
Intraoperative complications	4/12 (33.3%)	8/12 (66.7%)	0.250
Blood loss, mL *	133 ± 89	195 ± 103	0.360
Blood transfusions	1 (8.3%)	2 (2.4%)	0.587
Infusions, mL *	1658 ± 823	1836 ± 1076	0.134
Intraoperative lactates, mmol/L (average) *	3.28 ± 1.75	3.22 ± 1.56	0.918
Intraoperative lactates, mmol/L (highest value) *	3.99 ± 2.44	4.06 ± 2	0.715
Lymph node retrieval, n **	62 (59–77)	70 (61–81)	0.222
Intraoperative temperature			
Normothermia	2/14 (14.3%)	12/14 (85.7%)	0.015
Hypothermia	4/62 (6.5%)	58/62 (93.5%)	
Severe hypothermia	6/19 (31.6%)	13/19 (68.4%)	
Intraoperative TWA temperature ≤ 35 °C *	6/19 (31.6%)	13/19 (68.4%)	0.013
Intraoperative TWA temperature > 35 °C *	6/76 (7.9%)	70/76 (92.1%)	
Postoperative			
Postoperative WBC, ×10^9^/L *	7.9 ± 1.7	6.8 ± 2.4	0.183
Postoperative hemoglobin, g/dL *	11.5 ± 1.6	12.5 ± 1.6	0.106
Postoperative lactates, mmol/L *	3.05 ± 2.22	2.05 ± 1.02	0.179

* Average ± standard deviation. ** Median value (interquartile range). TWA, time-weighted average; aa-CCI, adjusted age-adjusted Charlson comorbidity index; ASA, American Society of Anesthesiologists; WBC, white blood cells.

**Table 3:** Differences in BMI, total operation time, abdominal and thoracic duration according to histologic diagnosis.

	Adenocarcinoma	Squamous Cell Carcinoma	*p Value*
Body mass index, kg/m^2^ *	24.2 ± 3.5	22.6 ± 4.7	0.267
Total operation (min) *	285 ± 53	255 ± 41	0.016
Abdominal stage (min) *	139 ± 50	114 ± 33	0.046
Thoracic stage (min) *	139 ± 39	145 ± 39	0.454
Lymph node retrieval (n) **	70 (61–84)	62 (61–72)	0.174

* Average ± standard deviation. ** Median value (interquartile range).

**Table 4:** Final multiple regression model for the outcome of anastomotic leakage in patients who underwent Ivor Lewis esophagectomy.

Variable	Multivariate Analysis		
	OR	95% CI	*p Value*
Gender (male vs. female)	–	–	–
Age (≥75 years)	–	–	–
Body mass index (Kg/m^2^)	–	–	–
Diagnosis (adenocarcinoma vs. SCC)	–	–	–
Neoadjuvant chemoradiotherapy	–	–	–
Severe hypothermia (Intraoperative TWA temperature < 35 °C)	5.385	1.502; 19.310	0.010

SCC, squamous cell carcinoma; TWA, time-weighted average.

**Table 5:** Final multiple regression model for the outcome of severe intraoperative hypothermia in patients who underwent Ivor Lewis esophagectomy.

Variable	Multivariate Analysis		
	OR	95% CI	*p Value*
Gender (male vs. female)	–	–	–
Age (≥75 years)	–	–	–
Body mass index (Kg/m^2^)	0.818	0.723; 0.926	0.001
Diagnosis (adenocarcinoma vs. SCC)	–	–	–
Neoadjuvant chemoradiotherapy	–	–	–
Intraoperative blood loss	–	–	–
Total intraoperative fluids (mL)	–	–	–
Operation time (min)	–	–	–

SCC, squamous cell carcinoma.

## Data Availability

Data are available upon request.

## Supplementary Materials

The following supporting information can be downloaded at https://www.mdpi.com/article/10.3390/cancers17071166/s1. Table S1: STROBE statement—Checklist of items that should be included in reports of cohort studies. Figure S1: Anesthesiology management according to the surgical approach.

## Appendix A

The OSR CCeR Collaborative Group: Lavinia A. Barbieri (Department of Gastrointestinal Surgery, IRCCS San Raffaele Scientific Institute, 20132 Milan, Italy), Silvia Battaglia (Department of Gastrointestinal Surgery, IRCCS San Raffaele Scientific Institute, 20132 Milan, Italy), Agnese Carresi (Department of Gastrointestinal Surgery, IRCCS San Raffaele Scientific Institute, 20132 Milan, Italy), Andrea Cossu (Department of Gastrointestinal Surgery, IRCCS San Raffaele Scientific Institute, 20132 Milan, Italy), Lorenzo Gozzini (Department of Gastrointestinal Surgery, IRCCS San Raffaele Scientific Institute, 20132 Milan, Italy), and Elio Treppiedi (Department of Gastrointestinal Surgery, IRCCS San Raffaele Scientific Institute, 20132 Milan, Italy).
